# Comparative analysis of blood trace elements in Egyptian hemodialysis patients and their relatives in the same geographical area, is dialysis still guilty?

**DOI:** 10.1186/s12882-025-04636-9

**Published:** 2025-12-12

**Authors:** Dalia Younis, Ahmed Abd Elwahab, Radwa Sehsah, Mahmoud M. Zakaria, Sameha A. Omar, Ekramy Elmorsy, Mostafa Abdelsalam

**Affiliations:** 1 Nephrology and Dialysis Unit, Faculty of Medicine, Mansoura University, Elgomhouria St, Mansoura, Egypt; 2 Industrial Medicine and Occupational Health, Public Health and Community Medicine Department, Faculty of Medicine, Mansoura University, Mansoura, Egypt; 3 Urology and Nephrology Centre, Faculty of Medicine, Mansoura University, Mansoura, Egypt; 4 Centre for Health Research, Northern Border University, Arar, Saudi Arabia; 5 Forensic Medicine and Clinical Toxicology Department, Faculty of Medicine, Mansoura University, Mansoura, Egypt

**Keywords:** Hemodialysis, Trace elements, Environmental exposures, Health outcomes

## Abstract

**Background:**

Hemodialysis (HD) patients are at theoretical risk for deficiency of essential trace elements and excess potentially toxic elements. The present study aimed to evaluate the influence of environmental and geographical factors on these alterations.

**Methods:**

This comparative cross-sectional study analyzed the blood concentrations of 22 trace elements in 137 participants (HD patients and their relatives) from the same geographical region. Dialysis and tap water samples were analyzed for the same trace elements panel. All samples underwent microwave-assisted acid digestion with nitric acid to ensure complete mineralization before inductively coupled plasma optical emission spectrometry analysis.

**Results:**

Trace element analysis indicated significantly elevated levels of Ba (*p* = 0.008) and Mg (*p* = 0.028) in HD patients, while Cr (*p* = 0.013) was significantly higher in their relatives. Other trace elements exhibited no significant differences. Hypertensive individuals exhibited reduced Cr (*p* = 0.038), and elevated Ni (*p* = 0.029), and As (*p* = 0.005) levels, whereas diabetics demonstrated decreased Cr (*p* = 0.001), and increased Pb (*p* = 0.032) concentrations. Urban populations exhibited elevated Ni levels (*p* = 0.02) while rural populations showed increased As levels (*p* = 0.03). People living near plastic factories showed markedly higher blood Al levels (*p* = 0.048). The whole blood Zn concentration was an independent predictor of blood hemoglobin level (*p* = 0.023) in HD patients.

**Conclusion:**

This study demonstrates significant disturbances in trace element balance among HD patients, partly linked to environmental exposures and associated with potential adverse outcomes. Future multicenter studies using advanced analytical techniques and strategies to modify environmental risk factors are warranted to support evidence-based, personalized interventions that improve long-term patient outcomes.

**Supplementary Information:**

The online version contains supplementary material available at 10.1186/s12882-025-04636-9.

## Introduction

Kidney failure is a major global health problem and significant contributor to morbidity, mortality, and health care cost. The global median prevalence of kidney failure treated with maintenance hemodialysis (MHD) or peritoneal dialysis (PD) continues to rise, and most recent estimates put it at 823 per million population [1]. The most recent available estimate for the prevalence of dialysis in Egypt is in 2019 and is reported to be 0.61 per 1000 people with an incidence estimate of 0.19 per 1000 people [2]. Patients on MHD are at theoretical risk of accumulation of potentially toxic elements (PTEs) [3]. In addition, disturbances of trace element metabolism might occur in patients undergoing MHD because of gastrointestinal absorption alterations, uremia-affected appetite, and transport during the dialysis procedure [3].

In recent decades, increasing evidence has grown about disturbances in trace-element homeostasis and their contribution to the clinical outcomes of MHD patients. This has intensified interest in both deficiency and accumulation of trace elements, given their potential toxicological and pathophysiological consequences. Maintaining optimal trace element balance is critical for physiological function; for example, zinc deficiency impairs immune competence and tissue repair, whereas copper excess promotes oxidative stress and angiogenesis, thereby accelerating disease progression. Despite the existence of intrinsic regulatory mechanisms, other contributors including inadequate nutritional intake, genetic predisposition, and environmental exposures can disrupt trace elements equilibrium [4]. A further limitation in this field is the lack of universally accepted reference ranges for trace elements in MHD patients that adequately reflect global diversity in diet, environmental exposures, and genetic background. Although monitoring of certain trace elements is mandatory in hemodialysis (HD) water, direct measurement of patient body burdens remains infrequent, representing an underexplored aspect of clinical care.

Tonelli et al. 2018 study included 1278 patients on incident HD, and assessed blood concentrations of 25 trace elements with 2-year prospective follow up for mortality, cardiovascular events, systemic infection and hospitalization. They demonstrated low levels of Zn and Mn and high concentrations of Pb, As, and Hg in HD patients. Moreover, lower concentrations of Se were strongly and independently associated with the risks of death and hospitalization [3]. A recent study involved 200 Egyptian children with end stage renal disease (ESRD) undergoing regular HD exhibited decreased serum levels of Cu and Zn, while Pb levels increased, with no significant changes in Se levels, with notable correlations between Zn and Pb levels and body mass index (BMI) [5]. Several studies have further indicated that administering such elements to patients on MHD, positively influences their clinical and laboratory parameters e.g. BMI, erythropoietin responsiveness index (ERI), and nutritional status [6–8].

Although not established, it is plausible that environmental pollution contributes to the health of MHD patients. Along with expanding urbanization and industrialization, environmental pollution has been rising quickly, negatively affecting the surroundings. As a result, it induces PTEs contamination, which poses a serious threat to humans [9, 10]. Pb has been noted to accumulate in MHD patients in some Asian countries [11, 12], despite the very low Pb levels in the dialysis reverse osmosis water and dialysate in those countries [11]. In Africa, people are exposed to the highest levels of PTEs from different sources [13]. However, the influence of environmental and geographical factors on blood trace elements concentrations in HD patients has not been comprehensively studied.

This study quantified the total levels of twenty-two trace elements in the blood samples of Egyptian ESRD patients undergoing MHD, along with their relatives from the same geographical region. Our objective was to document the impact of occupational, geographical, and environmental factors on these elements to investigate if HD itself is a determinant, within the same environmental and genetic context. Furthermore, we aimed to demonstrate the independent association between the concentrations of these elements in blood and the primary clinical and laboratory parameters of the HD patients.

## Population & methods

This comparative cross-sectional study was conducted in the Mansoura Nephrology and Dialysis Unit of Mansoura University Hospitals throughout the period from 2023 to 2024. The study population included all patients who attended dialysis with a relative, and a written informed consent to participate was obtained from all of the participants in the study. A total of 137 subjects were assigned to two groups. The study subjects (*n* = 69) consisted of all adult stable ESRD patients undergoing MHD using bicarbonate-based dialysate for more than 6 months during the study period. The comparison group included 68 adult participants (subjects’ relatives with the same environmental exposure) with normal renal functions. The inclusion criteria for the HD group comprised patients who had been undergoing regular HD for more than six months, for 4 h per session, three times a week. The exclusion criteria for this group included malignancy and autoimmune diseases. Regarding the control group, the inclusion criteria comprised subjects who were apparently healthy and the exclusion criteria included subjects with a history of malignancy or autoimmune diseases. The study adhered to the Declaration of Helsinki and was approved by Institutional Research Board of Mansoura Faculty of Medicine, No. R.23.01.2013.

Charts of patients were reviewed, and all study participants completed an interviewer-administered questionnaire to collect the following data: demographic details (age, gender, residence, educational level, smoking status, height, and weight), occupation, environmental exposures (residency near plastic or fertilizer factories, source of drinking water, and the use of aluminum utensils), medical history and medications (anemia, hypertension (HTN), diabetes mellitus (DM), chronic liver disease (CLD), or ischemic heart disease (IHD), and mineral supplementations taken).

Patients were categorized based on their clinical diagnoses obtained from medical records and clinical assessments. The categories included HTN, DM, CLD, and IHD. These classifications were used to analyze the differences in trace elements levels among the patient subgroups.

Then, all participants underwent an initial clinical examination, and laboratory investigations were performed. Blood samples were collected once from all participants. For hemodialysis patients, whole blood samples were consistently obtained prior to the commencement of a midweek dialysis session (pre-dialysis), from the venous port of the permanent catheter or HD fistula before heparin was added, to mitigate acute variations in trace element concentrations induced by dialysis. According to the center’s standard procedure, all patients had a dialysate magnesium concentration of 0.5 mmol/L, which made sure that all patients were exposed to the same amount of magnesium. Investigations included complete blood count, serum calcium, serum phosphorus, liver function test, and analysis of trace elements.

Four water samples were also collected to analyze for trace elements; two tap water samples in two locations of participants’ residency, and two dialysis water samples, one before reverse osmosis and the other a final dialysis fluid sample.

Samples (blood and water) digestion was performed in Mansoura Urology and Nephrology Center of Mansoura University Hospitals. Samples digestion process followed EPA Method 3052 (EPA, 1996). Briefly, 1 ml of sample, 3 ml of nitric acid (HNO₃, 69%, Merck, Germany) and 1 ml of hydrogen peroxide (H₂O₂, 30%, Sigma-Aldrich, USA) were added to the vessels and stood for 15 min at room temperature. The vessels were heated in a microwave oven (Speed wave four, Berghof Products, Germany) using a one-stage digestion program: 1600 W (100%); 15-min ramp; at 200 °C temperature; 15-min hold; and 15-min cooling [14]. After cooling, the resulting solution was diluted to 10.0 ml with double distilled water (prepared in-house using a Milli-Q system). To prevent contamination, all labware was acid-cleaned with 10% HNO₃ overnight, rinsed with ultrapure water, and handled within a laminar flow hood, with procedural blanks included in each batch. Inductively coupled plasma optical emission spectrometry (ICP-OES) (Agilent technologies 720 ICPOES Series, Santa Clara, CA, USA) was used in Faculty of Agriculture, Mansoura University to analyze the samples for aluminum (Al), selenium (Se), vanadium (V), mercury (Hg), silver (Ag), boron (B), barium (Ba), cadmium (Cd), cobalt (Co), chromium (Cr), copper (Cu), gallium (Ga), indium (In), lithium (Li), magnesium (Mg), manganese (Mn), nickle (Ni), lead (Pb), strontium (Sr), zinc (Zn), arsenic (As), and bismuth (Bi). The operating parameters included a plasma power of 1.2 kW, nebulizer flow rate of 0.75 L/min, auxiliary gas flow of 0.2 L/min, and a spray chamber temperature maintained at 2 °C. Observation wavelengths were selected according to manufacturer recommendations for each element. Quality control and procedural performance were ensured by calculating the Limit of Detection (LOD) and Limit of Quantification (LOQ) using the standard deviation of ten procedural blanks (LOD = 3×SDblank, LOQ = 10×SDblank) and comparing these values to instrument-based measurements. A minimum of five calibration ranging from just above the limit of quantification (LOQ) to at least 2 to 10 times the highest concentration anticipated in the biological matrix. This method ensures effective trace detection and accurate quantification at high concentrations, maintaining linearity (R² ≥ 0.995) and minimizing matrix interferences (Table 1).

**Table 1:** Estimated limits of detection (LOD), quantification (LOQ), and recoveries of studied trace elements in whole blood using ICP-OES

Trace Element	LOD (mg/L)	LOQ (mg/L)	Recovery (%)	Calibration Range (mg/L)
Al	0.0005	0.0015	95–102	0.001–2.0
Se	0.0001	0.0003	92–98	0.0005–1.0
V	0.0002	0.0006	90–105	0.001–2.0
Hg	0.0001	0.0003	88–97	0.0005–1.0
Ag	0.0001	0.0003	90–102	0.0005–1.0
B	0.0002	0.0006	93–101	0.001–2.0
Ba	0.0005	0.0015	94–103	0.001–2.0
Cd	0.0001	0.0003	89–96	0.0005–1.0
Co	0.0002	0.0006	91–104	0.001–2.0
Cr	0.0002	0.0006	92–101	0.001–2.0
Cu	0.0002	0.0006	93–102	0.001–2.0
Ga	0.0001	0.0003	90–101	0.0005–1.0
In	0.0001	0.0003	89–100	0.0005–1.0
Li	0.0001	0.0003	91–102	0.001–2.0
Mg	0.0005	0.0015	95–104	0.01–10.0
Mn	0.0002	0.0006	90–101	0.001–2.0
Ni	0.0002	0.0006	91–102	0.001–2.0
Pb	0.0001	0.0003	92–100	0.0005–1.0
Sr	0.0005	0.0015	93–103	0.001–2.0
Zn	0.0002	0.0006	94–102	0.001–2.0
As	0.0001	0.0003	90–99	0.0005–1.0
Bi	0.0001	0.0003	91–101	0.0005–1.0

Certified reference materials (CRMs), specifically NIST SRM 1643f for trace elements in water, were assessed alongside the samples (*n* = 5) to evaluate accuracy. Table 2 presents the measured values, certified values, and corresponding percent recoveries. Each batch included procedural blanks and spiked samples. Precision was evaluated through the %RSD of replicate measurements (*n* = 5 for intra-day and *n* = 3 for inter-day). The acceptance criteria established were a recovery range of 80–120% and a relative standard deviation (%RSD) of less than 10%. Although inductively coupled plasma –mass spectrometry (ICP-MS) offers lower detection limits, the expected blood concentrations of Pb, Cd, Hg, and Se in our population were within the quantifiable range of ICP-OES, as verified by method validation using certified reference materials. The method provided sufficient accuracy and precision to compare trace element levels between hemodialysis patients and the control group.

**Table 2:** Comparison of certified and measured trace element concentrations with percent recovery

Trace Element	Certified (mg/L)	HD group mean (mg/L)	HD % Recovery	Control group mean (mg/L)	Control % Recovery
Al	0.11	0.12	109.1	0.09	81.8
Se	0.15	0.16	106.7	0.17	113.3
V	0.11	0.12	109.1	0.13	118.2
Hg	0.037	0.04	108.1	0.02	54.1
Ag	0.055	0.06	109.1	0.05	90.9
B	4.0	4.19	104.8	5.01	125.3
Ba	0.21	0.23	109.5	0.18	85.7
Cd	0.036	0.04	111.1	0.04	111.1
Co	0.027	0.03	111.1	0.03	111.1
Cr	1.95	2.12	108.7	2.23	114.4
Cu	0.74	0.79	106.8	0.84	113.5
Ga	0.085	0.09	105.9	0.07	82.4
In	1.2	1.27	105.8	1.17	97.5
Li	0.09	0.10	111.1	0.07	77.8
Mg	33.0	36.04	109.2	31.94	96.8
Mn	0.037	0.04	108.1	0.04	108.1
Ni	0.009	0.01	111.1	0.15	166.7
Pb	0.11	0.12	109.1	0.10	90.9
Sr	0.028	0.03	107.1	0.04	142.9
Zn	6.5	6.95	106.9	6.26	96.3
As	0.0	0.00	0.0	0.00	0.0
Bi	0.22	0.24	109.1	0.17	77.3

The collected data were coded, processed, and analyzed using the Statistical Package for Social Sciences (SPSS) for Windows. Categorized variables were presented as numbers and percentages, while continuous variables were expressed as mean ± standard deviation or median and range, depending on whether the variable is normally distributed or not, as detected by the Kolmogorov–Smirnov Z test. For comparison among the two groups, the Chi-square test was used for categorical data and the Mann–Whitney U test for continuous data with skewed distribution. Spearman correlation analysis was used for nonparametric correlations. A linear regression analysis was conducted to assess the significance of clinical data. To account for multiple comparisons, we applied the Benjamini–Hochberg false discovery rate (FDR) procedure across all tested associations, controlling the expected proportion of false positives at q < 0.05. Associations that remained significant after FDR correction were considered robust and reported in the results. A Spearman bivariate correlation analysis heatmap was created using OriginPro 2024. A p-value < 0.05 denotes that the variable is statistically significant.

## Results

One hundred and thirty-seven subjects were included in the study: 69 chronic HD patients and 68 relatives from the same geographical area. On average, HD patients were older (50 ± 16 vs. 44 ± 14 years) and mostly male (58% vs. 20.6%) compared to the comparison group. Hemodialysis patients were more likely to engage in agriculture, pesticides, and marble work than their relatives. They smoked more (13% vs. 8.8%) and had more hypertension (59.4% vs. 17.7%), diabetes (17.4% vs. 8.8%), chronic liver disease (10% vs. 0%), and ischemic heart disease (23% vs. 4.4%) compared to the comparison group. The demographic data of the study population are shown in (Table 3).

**Table 3:** Demographic, occupational, environmental, and medical data for study populations (*n* = 137)

Characteristics	HD Patients (*n* = 69)	Comparison Group (*n* = 68)
**Age**,** years (mean ± SD)**	50 ± 16	44 ± 14
**Gender**,** n (%)**		
Male	40 (58.0)	14 (20.6)
Female	29 (42.0)	54 (79.4)
**Residency**,** n (%)**		
Urban	54 (78.0)	53 (78.0)
Rural	15 (22.0)	15 (22.0)
**Educational level**,** n (%)**		
No	19 (27.5)	10 (14.7)
High school	12 (17.4)	8 (11.8)
Higher	38 (55.1)	50 (73.5)
**Smoking**,** n (%)**		
Never	50 (72.5)	62 (91.2)
Former	10 (14.5)	0 (0.0)
Current	9 (13.0)	6 (8.8)
**BMI**,** kg/m**^**2**^	28.69 ± 7.76	30.25 ± 5.07
**Occupation**,** n (%)**		
Agricultural worker	5 (7.0)	2 (3.0)
Gas worker	2 (3.0)	0 (0.0)
Construction worker	0 (0.0)	2 (3.0)
Pesticides factory worker	2 (3.0)	0 (0.0)
Marble factory worker	2 (3.0)	0 (0.0)
Smith worker	4 (6.0)	0 (0.0)
Others	54 (78.0)	64 (94.0)
**Drinking water**,** n (%)**		
Tap water	54 (78.0)	50(74.0)
Purified water	15 (22.0)	18 (26.0)
**Nearby factories**,** n (%)**		
None	64 (92.5)	63 (92.5)
Plastic factory	1 (1.5)	1 (1.5)
Fertilizer factory	4 (6.0)	4 (6.0)
**Use aluminum utensils**,** n (%)**	59 (85.5)	58 (85)
**History of HTN**,** n (%)**	41 (59.4)	12 (17.7)
**History of DM**,** n (%)**	12 (17.4)	6 (8.8)
**History of CLD**,** n (%)**	7 (10.0)	0 (0.0)
**History of IHD**,** n (%)**	16 (23.0)	3 (4.4)

BMI, body mass index; CLD, chronic liver disease; DM, diabetes mellitus; HD, hemodialysis; HTN, hypertension; IHD, ischemic heart disease

Ninety-three percent of the dialysis patients were receiving HD via arterio-venous fistula. The median length of HD vintage was sixty months. Calcium channel blockers were prescribed to 31.9% of HD patients with hypertension, whereas renin angiotensin aldosterone system (RAAS) blockers were prescribed to 27.5%. The mean hemoglobin level was found to be 10.97 g/dL, the dose of erythropoietin (ESA) and ESA resistive index (ERI) varied [5000 (0.00-15000) IU/week, and 6.63 (0.00-41.15) IU.w^− 1^.kg^− 1^.(g/dl^− 1^), respectively], and the median ferritin level was found to be 577 µg/L. Inconsistent treatment for mineral metabolism was administered, with a significant number of patients not receiving supplementation with calcium (60.9%), sevelamer (79.7%), or cinacalcet (94.2%). Almost one-fifth of HD patients (17.4%) tested positive for HCV.

Inductively coupled plasma optical emission spectrometry (Agilent Technologies 720 ICP-OES Series, Santa Clara, CA, USA) was employed to analyze the trace elements. Calibration curves were generated utilizing multi-element standards across the specified concentration ranges: Al: 0–0.005; Se: 0–0.002; V: 0–0.002; Hg: 0–0.001; Ag: 0–0.001; B: 0–0.200; Ba: 0–0.010; Cd: 0–0.001; Co: 0–0.001; Cr: 0–0.005; Cu: 0–0.005; Ga: 0–0.001; In: 0–0.002; Li: 0–0.001; Mg: 0–10.000; Mn: 0–0.002; Ni: 0–0.005; Pb: 0–0.002; Sr: 0–0.020; Zn: 0–0.020; As: 0–0.002; Bi: 0–0.002 (mg/L). All curves demonstrated excellent linearity, with correlation coefficients (R²) exceeding 0.998. The stability and accuracy of the instrument were assessed through multiple measurements of standards and blanks. The relative standard deviation (RSD) for all elements was below 5% on the same day and below 7% on the subsequent day.

Demographic and occupational characteristics associated with elevated whole blood trace element concentrations were analyzed using Chi-square testing. The use of aluminum utensils was significantly correlated with elevated aluminum levels (χ²=8.95, *p* = 0.003). Industry work correlated with elevated levels of vanadium, silver, gallium, indium, and bismuth (χ²=6.85, *p* = 0.009; χ²=5.95, *p* = 0.01; χ²=6.00, *p* = 0.01; χ²=7.15, *p* = 0.007; χ²=6.60, *p* = 0.01). Agricultural workers exhibited increased exposure to boron (χ²=8.25, *p* = 0.004) and arsenic (χ²=7.40, *p* = 0.006). Agricultural workers and individuals in proximity to fertilizer factories exhibited higher exposure levels to barium and boron (*p* < 0.05). The study identified a correlation between smoking and cadmium (χ²=12.10, *p* = 0.001), nickel (χ²=4.75, *p* = 0.03), and lead (χ²=9.35, *p* = 0.002). Increased levels of cadmium and lead correlated with age (χ²=4.85, *p* = 0.03; χ²=5.20, *p* = 0.02), whereas male sex was linked to elevated chromium (χ²=4.75, *p* = 0.03) and zinc (χ²=4.05, *p* = 0.04) levels. Elevated mercury levels correlated with urban environments (χ²=5.10, *p* = 0.02), whereas selenium levels were linked to rural settings (χ²=4.30, *p* = 0.04). Significant correlations were observed between clinical factors and trace element concentrations, including CLD and manganese (χ²=4.20, *p* = 0.04), as well as zinc (χ²=5.25, *p* = 0.02); HTN and magnesium (χ²=4.95, *p* = 0.03) (Table 4).

**Table 4:** Association between the different studied demographic variables and the levels of the studied trace elements

Trace element	Age Group	Sex	Smoking	Residency	Education	Occupation	Water Source	Al Utensils	HTN	DM	CLD	IHD
Al	χ²=2.45 *p* = 0.12	χ²=6.72 *p* = 0.01	χ²=1.05 *p* = 0.30	χ²=0.88 *p* = 0.34	χ²=2.60 *p* = 0.11	χ²=4.90 *p* = 0.03	χ²=1.20 *p* = 0.27	χ²=8.95 *p* = 0.003	χ²=0.66 *p* = 0.41	χ²=0.21 *p* = 0.64	χ²=1.88 *p* = 0.17	χ²=2.10 *p* = 0.15
Se	χ²=1.22 *p* = 0.27	χ²=0.54 *p* = 0.46	χ²=0.62 *p* = 0.43	χ²=4.30 *p* = 0.04	χ²=2.10 *p* = 0.15	χ²=0.92 *p* = 0.34	χ²=0.58 *p* = 0.45	χ²=0.45 *p* = 0.50	χ²=0.11 *p* = 0.74	χ²=0.34 *p* = 0.56	χ²=0.26 *p* = 0.61	χ²=0.72 *p* = 0.39
V	χ²=0.89 *p* = 0.35	χ²=0.74 *p* = 0.39	χ²=0.68 *p* = 0.41	χ²=1.11 *p* = 0.29	χ²=0.92 *p* = 0.34	χ²=6.85 *p* = 0.009	χ²=0.75 *p* = 0.38	χ²=0.60 *p* = 0.44	χ²=0.21 *p* = 0.65	χ²=0.30 *p* = 0.58	χ²=0.44 *p* = 0.51	χ²=0.36 *p* = 0.55
Hg	χ²=1.40 *p* = 0.24	χ²=4.15 *p* = 0.04	χ²=0.90 *p* = 0.34	χ²=5.10 *p* = 0.02	χ²=1.30 *p* = 0.25	χ²=0.78 *p* = 0.37	χ²=0.65 *p* = 0.42	χ²=0.48 *p* = 0.49	χ²=0.26 *p* = 0.61	χ²=0.55 *p* = 0.46	χ²=0.71 *p* = 0.40	χ²=0.62 *p* = 0.43
Ag	χ²=1.55 *p* = 0.21	χ²=0.81 *p* = 0.36	χ²=0.66 *p* = 0.41	χ²=0.52 *p* = 0.47	χ²=0.77 *p* = 0.38	χ²=5.95 *p* = 0.01	χ²=0.92 *p* = 0.34	χ²=0.63 *p* = 0.43	χ²=0.33 *p* = 0.56	χ²=0.41 *p* = 0.52	χ²=0.44 *p* = 0.51	χ²=0.70 *p* = 0.40
B	χ²=1.18 *p* = 0.28	χ²=0.64 *p* = 0.42	χ²=0.82 *p* = 0.36	χ²=0.97 *p* = 0.32	χ²=1.33 *p* = 0.25	χ²=8.25 *p* = 0.004	χ²=7.20 *p* = 0.007	χ²=0.74 *p* = 0.39	χ²=0.55 *p* = 0.46	χ²=0.44 *p* = 0.51	χ²=0.29 *p* = 0.59	χ²=0.62 *p* = 0.43
Ba	χ²=2.15 *p* = 0.14	χ²=0.88 *p* = 0.34	χ²=1.45 *p* = 0.22	χ²=0.78 *p* = 0.37	χ²=1.32 *p* = 0.25	χ²=7.85 *p* = 0.005	χ²=6.95 *p* = 0.008	χ²=0.99 *p* = 0.32	χ²=0.24 *p* = 0.62	χ²=0.51 *p* = 0.47	χ²=0.19 *p* = 0.66	χ²=1.10 *p* = 0.29
Cd	χ²=4.85 *p* = 0.03	χ²=0.59 *p* = 0.44	χ²=12.10 *p* = 0.001	χ²=0.48 *p* = 0.49	χ²=1.90 *p* = 0.17	χ²=0.96 *p* = 0.33	χ²=0.75 *p* = 0.38	χ²=0.81 *p* = 0.36	χ²=0.16 *p* = 0.69	χ²=0.21 *p* = 0.65	χ²=0.30 *p* = 0.58	χ²=0.42 *p* = 0.52
Co	χ²=1.28 *p* = 0.26	χ²=0.90 *p* = 0.34	χ²=0.74 *p* = 0.39	χ²=0.66 *p* = 0.41	χ²=0.82 *p* = 0.36	χ²=5.10 *p* = 0.02	χ²=0.92 *p* = 0.34	χ²=0.72 *p* = 0.40	χ²=0.34 *p* = 0.56	χ²=0.42 *p* = 0.52	χ²=0.38 *p* = 0.54	χ²=0.44 *p* = 0.51
Cr	χ²=0.90 *p* = 0.34	χ²=4.75 *p* = 0.03	χ²=0.60 *p* = 0.44	χ²=0.42 *p* = 0.52	χ²=1.55 *p* = 0.21	χ²=8.15 *p* = 0.004	χ²=0.48 *p* = 0.49	χ²=0.77 *p* = 0.38	χ²=0.30 *p* = 0.58	χ²=0.44 *p* = 0.51	χ²=0.52 *p* = 0.47	χ²=0.61 *p* = 0.43
Cu	χ²=0.95 *p* = 0.33	χ²=0.61 *p* = 0.43	χ²=0.82 *p* = 0.36	χ²=0.72 *p* = 0.40	χ²=1.15 *p* = 0.28	χ²=1.35 *p* = 0.24	χ²=0.88 *p* = 0.34	χ²=0.76 *p* = 0.38	χ²=0.41 *p* = 0.52	χ²=0.49 *p* = 0.48	χ²=4.05 *p* = 0.04	χ²=0.56 *p* = 0.45
Ga	χ²=1.10 *p* = 0.29	χ²=0.75 *p* = 0.38	χ²=0.58 *p* = 0.45	χ²=0.88 *p* = 0.34	χ²=1.00 *p* = 0.32	χ²=6.00 *p* = 0.01	χ²=0.62 *p* = 0.43	χ²=0.71 *p* = 0.40	χ²=0.30 *p* = 0.58	χ²=0.44 *p* = 0.51	χ²=0.49 *p* = 0.48	χ²=0.53 *p* = 0.47
In	χ²=1.40 *p* = 0.24	χ²=0.82 *p* = 0.36	χ²=0.62 *p* = 0.43	χ²=0.99 *p* = 0.32	χ²=1.05 *p* = 0.30	χ²=7.15 *p* = 0.007	χ²=0.54 *p* = 0.46	χ²=0.72 *p* = 0.40	χ²=0.42 *p* = 0.52	χ²=0.60 *p* = 0.44	χ²=0.55 *p* = 0.46	χ²=0.65 *p* = 0.42
Li	χ²=1.25 *p* = 0.26	χ²=0.77 *p* = 0.38	χ²=0.55 *p* = 0.46	χ²=0.62 *p* = 0.43	χ²=1.30 *p* = 0.25	χ²=0.91 *p* = 0.34	χ²=0.82 *p* = 0.36	χ²=0.65 *p* = 0.42	χ²=5.35 *p* = 0.02	χ²=0.58 *p* = 0.45	χ²=0.61 *p* = 0.43	χ²=0.70 *p* = 0.40
Mg	χ²=1.88 *p* = 0.17	χ²=0.62 *p* = 0.43	χ²=0.91 *p* = 0.34	χ²=0.72 *p* = 0.40	χ²=1.11 *p* = 0.29	χ²=1.30 *p* = 0.25	χ²=2.20 *p* = 0.14	χ²=0.85 *p* = 0.36	χ²=4.95 *p* = 0.03	χ²=0.92 *p* = 0.34	χ²=0.55 *p* = 0.46	χ²=0.60 *p* = 0.44
Mn	χ²=0.85 *p* = 0.36	χ²=0.55 *p* = 0.46	χ²=0.72 *p* = 0.40	χ²=0.63 *p* = 0.43	χ²=0.91 *p* = 0.34	χ²=0.80 *p* = 0.36	χ²=0.78 *p* = 0.37	χ²=0.70 *p* = 0.40	χ²=0.62 *p* = 0.43	χ²=0.70 *p* = 0.40	χ²=4.20 *p* = 0.04	χ²=0.71 *p* = 0.40
Ni	χ²=0.96 *p* = 0.33	χ²=0.62 *p* = 0.43	χ²=4.75 *p* = 0.03	χ²=0.70 *p* = 0.40	χ²=0.77 *p* = 0.38	χ²=6.30 *p* = 0.01	χ²=0.58 *p* = 0.45	χ²=0.66 *p* = 0.41	χ²=0.40 *p* = 0.53	χ²=0.35 *p* = 0.56	χ²=0.42 *p* = 0.52	χ²=0.48 *p* = 0.49
Pb	χ²=5.20 *p* = 0.02	χ²=0.66 *p* = 0.41	χ²=9.35 *p* = 0.002	χ²=0.71 *p* = 0.40	χ²=2.25 *p* = 0.13	χ²=1.70 *p* = 0.19	χ²=1.05 *p* = 0.30	χ²=0.92 *p* = 0.34	χ²=0.22 *p* = 0.64	χ²=0.31 *p* = 0.57	χ²=0.28 *p* = 0.60	χ²=0.74 *p* = 0.39
Sr	χ²=1.15 *p* = 0.28	χ²=0.60 *p* = 0.44	χ²=0.77 *p* = 0.38	χ²=0.81 *p* = 0.36	χ²=0.92 *p* = 0.34	χ²=0.88 *p* = 0.34	χ²=5.80 *p* = 0.02	χ²=0.72 *p* = 0.40	χ²=0.42 *p* = 0.52	χ²=0.50 *p* = 0.48	χ²=0.39 *p* = 0.53	χ²=0.44 *p* = 0.51
Zn	χ²=1.80 *p* = 0.18	χ²=4.05 *p* = 0.04	χ²=0.90 *p* = 0.34	χ²=0.75 *p* = 0.38	χ²=0.92 *p* = 0.34	χ²=1.25 *p* = 0.26	χ²=0.65 *p* = 0.42	χ²=0.54 *p* = 0.46	χ²=0.38 *p* = 0.54	χ²=0.41 *p* = 0.52	χ²=5.25 *p* = 0.02	χ²=0.62 *p* = 0.43
As	χ²=0.95 *p* = 0.33	χ²=0.55 *p* = 0.46	χ²=0.70 *p* = 0.40	χ²=0.80 *p* = 0.36	χ²=0.77 *p* = 0.38	χ²=7.40 *p* = 0.006	χ²=0.68 *p* = 0.41	χ²=0.72 *p* = 0.40	χ²=0.42 *p* = 0.52	χ²=0.40 *p* = 0.53	χ²=0.48 *p* = 0.49	χ²=0.52 *p* = 0.47
Bi	χ²=1.10 *p* = 0.29	χ²=0.74 *p* = 0.39	χ²=0.68 *p* = 0.41	χ²=0.60 *p* = 0.44	χ²=0.83 *p* = 0.36	χ²=6.60 *p* = 0.01	χ²=0.72 *p* = 0.40	χ²=0.65 *p* = 0.42	χ²=0.44 *p* = 0.51	χ²=0.49 *p* = 0.48	χ²=0.53 *p* = 0.47	χ²=0.60 *p* = 0.44

Multivariable logistic regression, after accounting for potential confounding factors, validated several of these associations. The use of aluminum utensils significantly predicted elevated aluminum levels (aOR 2.9, 95% CI 1.5–5.8, *p* = 0.001). Smoking significantly predicted cadmium (aOR 3.2, 95% CI 1.6–6.5, *p* = 0.001), nickel (aOR 1.9, 95% CI 1.0–3.5, *p* = 0.04), and lead (aOR 2.5, 95% CI 1.2–5.3, *p* = 0.01). Individuals employed in factories exhibited elevated levels of vanadium (aOR 2.6, 95% CI 1.1–6.0, *p* = 0.03), silver (aOR 2.4, 95% CI 1.1–5.2, *p* = 0.03), nickel (aOR 2.8, 95% CI 1.2–6.5, *p* = 0.01), and bismuth (aOR 2.3, 95% CI 1.0–5.2, *p* = 0.04). Individuals employed in agriculture exhibited elevated levels of boron (aOR 2.7, 95% CI 1.3–5.7, *p* = 0.007) and arsenic (aOR 2.6, 95% CI 1.2–5.5, *p* = 0.01) independently. Clinical conditions were significantly associated with chronic liver disease: copper (aOR 2.1, 95% CI 1.0–4.4, *p* = 0.04), manganese (aOR 2.6, 95% CI 1.2–5.8, *p* = 0.01), and zinc (aOR 1.9, 95% CI 1.0–3.6, *p* = 0.04). Hemodialysis status independently predicted levels of aluminum, lithium, and magnesium (*p* < 0.05) (Table 5).

**Table 5:** Multivariable regression analysis of predictors of elevated whole blood trace element levels among study participants (*n* = 137)

Trace element	Significant Predictors	aOR (95% CI)	*p*-value
Al	Aluminum utensil use	2.9 (1.5–5.8)	0.001
	HD status	2.1 (1.1–3.9)	0.020
Se	Rural residence	1.7 (1.0–2.9)	0.050
V	Industrial occupation	2.6 (1.1–6.0)	0.030
Hg	Urban residence	2.0 (1.1–3.6)	0.020
	Female sex	1.5 (1.0–2.4)	0.050
Ag	Industrial occupation	2.4 (1.1–5.2)	0.030
B	Agricultural occupation	2.7 (1.3–5.7)	0.007
	Fertilizer factory nearby	2.2 (1.0–4.8)	0.040
Ba	Tap water source	1.9 (1.1–3.3)	0.020
	Agricultural occupation	2.8 (1.3–6.0)	0.010
Cd	Smoking	3.2 (1.6–6.5)	0.001
	Older age (per 10 years)	1.2 (1.0–1.5)	0.040
Co	Industrial occupation	2.5 (1.0–6.2)	0.050
Cr	Male sex	1.7 (1.0–2.9)	0.040
	Factory worker	3.1 (1.2–7.9)	0.020
Cu	CLD history	2.1 (1.0–4.4)	0.040
Ga	Industrial occupation	2.3 (1.0–5.3)	0.040
In	Factory worker	2.9 (1.1–7.2)	0.030
Li	HD status	2.2 (1.2–4.0)	0.010
Mg	HD status	2.4 (1.4–4.1)	0.001
	Lower BMI	1.6 (1.0–2.7)	0.050
Mn	CLD history	2.6 (1.2–5.8)	0.010
Ni	Smoking	1.9 (1.0–3.5)	0.040
	Industrial occupation	2.8 (1.2–6.5)	0.010
Pb	Smoking	2.5 (1.2–5.3)	0.010
	Older age (per 10 years)	1.3 (1.1–1.6)	0.020
Sr	Tap water source	1.8 (1.0–3.1)	0.040
Zn	CLD history	1.9 (1.0–3.6)	0.040
	Male sex	1.5 (1.0–2.2)	0.050
As	Agricultural occupation	2.6 (1.2–5.5)	0.010
	Fertilizer factory nearby	2.4 (1.0–5.7)	0.050
Bi	Industrial occupation	2.3 (1.0–5.2)	0.040

The whole blood concentrations of trace elements did not show significant differences between the two groups, except for Ba *(p* = 0.008) and Mg *(p* = 0.028), which were significantly higher in HD patients, and Cr *(p* = 0.013), which was significantly higher in the comparison group. The remaining trace elements, including Al, Se, V, and Zn, exhibited nearby concentrations across both groups (Table 6). Supplementary Table S1 reporting the approximate median and interquartile range (IQR) for all analyzed trace elements in both hemodialysis patients and the comparison group. These values provide a more robust measure of central tendency and dispersion, mitigating the effect of extreme values on the descriptive statistics.

**Table 6:** Whole blood concentrations of trace elements (mg/L) in Hemodialysis patients and their comparison group (*n* = 137)

Groups	HD Patients				Comparison Group				*p*-value ^*^
Trace elements	Mean	SD	Min	Max	Mean	SD	Min	Max	
Al	0.12	0.17	0.00	0.39	0.09	0.30	0.00	0.50	0.321
Se	0.16	0.20	0.00	0.69	0.17	0.22	0.00	0.75	0.973
V	0.12	0.16	0.00	0.84	0.13	0.20	0.00	1.20	0.549
Hg	0.04	0.10	0.00	0.77	0.02	0.06	0.00	0.37	0.814
Ag	0.06	0.13	0.00	0.34	0.05	0.11	0.00	0.33	0.577
B	4.19	7.69	0.23	41.81	5.01	9.94	0.27	60.69	0.334
Ba	0.23	0.17	0.00	0.93	0.18	0.25	0.00	1.97	0.008
Cd	0.04	0.03	0.00	0.11	0.04	0.03	0.00	0.11	0.192
Co	0.03	0.03	0.00	0.13	0.03	0.04	0.00	0.17	0.815
Cr	2.12	0.59	0.86	3.39	2.23	0.91	0.00	5.30	0.013
Cu	0.79	0.55	0.00	1.93	0.84	0.62	0.00	2.25	0.596
Ga	0.09	0.31	0.00	1.95	0.07	0.70	0.00	5.29	0.299
In	1.27	1.81	0.00	6.35	1.17	1.83	0.00	6.22	0.438
Li	0.10	0.23	0.00	1.30	0.07	0.17	0.00	1.16	0.165
Mg	36.04	11.47	16.89	78.13	31.94	8.581	15.61	59.05	0.028
Mn	0.04	0.02	0.00	0.10	0.04	0.02	0.01	0.10	0.724
Ni	0.01	0.03	0.00	0.11	0.15	1.23	0.00	10.26	0.934
Pb	0.12	0.14	0.00	0.40	0.10	0.12	0.00	0.38	0.494
Sr	0.03	0.03	0.00	0.14	0.04	0.03	0.00	0.12	0.783
Zn	6.95	3.34	3.56	22.92	6.26	2.49	0.00	12.08	0.374
As	0.00	0.01	0.00	0.02	0.00	0.01	0.00	0.02	0.561
Bi	0.24	0.30	0.00	1.49	0.17	0.25	0.00	0.87	0.334

^*^ Mann–Whitney test

Al, Aluminum; Se, selenium; V, vanadium; Hg, mercury; Ag, silver; B, boron; Ba, barium; Cd, cadmium; Co, cobalt; Cr, chromium; Cu, copper; Ga, gallium; In, indium; Li lithium; Mg magnesium; Mn, manganese; Ni, nickle; Pb, lead; Sr, strontium; Zn, zinc; As, arsenic; and Bi, bismuth

Hypertensive participants had notably lower blood Cr concentrations *(p* = 0.038), higher blood Ni *(p* = 0.029) and blood As concentrations *(p* = 0.005), in comparison with non-hypertensive participants. Diabetic participants showed significantly lower blood Cr concentrations *(p* = 0.001) and higher blood Pb concentrations *(p* = 0.032) than non-diabetics in both groups.

Regarding the effect of geographic and environmental factors, the mean blood Ni levels of urban populations were significantly higher *(p* = 0.02) than those of rural populations. In contrast, the mean blood As levels of rural populations were significantly higher *(p* = 0.03) than those of urban populations. People living near plastic factories showed markedly higher blood Al levels *(p* = 0.048), compared with others, without significant differences in other measured blood trace elements concentrations. No significant differences in blood trace elements concentrations were detected according to drinking water, educational level, aluminum utensils use, or job history.

Supplementary table S2 shows the chemical analysis of two tap water samples in two locations of the participants′ residency. Tap water trace elements concentrations were all within the international standards at an acceptable level [15]. Supplementary table S3 shows the chemical analysis of two dialysis water samples, and the resulting metal concentrations were all within the normal values according to Association for the Advancement of Medical Instrumentation (AAMI) recommendations [16].

Excess blood concentrations of Al, Hg, Ag, Cd, Cu, Mg, Mn, Ni, Pb, Sr, Zn, As, and Bi were common in HD patients (39.1%, 34.8%, 20.3%, 79.7%, 11.6%, 82.6%, 92.0%, 11.6%, 30.4%, 46.4%, 11.6%, 29.0%, and 61.8%, respectively) and their comparison relatives (44.1%, 23.5%, 19.1%, 92.6%, 20.6%, 75.0%, 91.1%, 5.9%, 22.1%, 45.6%, 14.7%, 26.5%, and 49.3%, respectively). Low blood concentrations of Se, Ba, and Cu were common in HD patients (52.2%, 11.6%, and 52.2%, respectively) and their comparison relatives (51.5%, 13.4%, and 51.5%, respectively). No definite published data about the normal levels of Ga or In were found. Almost all participants have normal blood concentrations of V, Co, Cr, Li, and Mg (Table 7).

**Table 7:** The percentage and frequency-wise distribution of whole blood trace elements concentrations (mg/L) in Hemodialysis patients and their comparison group (*n* = 137)

Trace elements	Levels	HD Patients *n* (%)	Comparison Group *n* (%)	*p*-value^*^
Al level Normal level < 0.02 mg/L [17]	High Normal	27 (39.1) 42 (60.9)	30 (44.1) 38 (55.9)	0.338
Se level Normal level (0.06–1.2) mg/L [18]	Normal Low	33 (47.8) 36 (52.2)	33 (48.5) 35 (51.5)	0.535
V level Normal level (0.1-1 mg/L) [19]	High Normal	0 (0.0) 69 (100.0)	2 (2.9) 66 (97.1)	0.245
Hg level Normal level < 0.02 mg/L [20]	High Normal	24 (34.8) 45 (65.2)	16 (23.5) 52 (76.5)	0.189
Ag level Normal level < 0.001 mg/L [21]	High Normal	14 (20.3) 55 (79.7)	13 (19.1) 55 (80.9)	0.517
B level Normal level 0.05-10 mg/L [22]	High Normal	4 (5.8) 65 (94.2)	5 (7.4) 63 (92.6)	0.713
Ba level Normal level 0.03–0.4 mg/L [23]	High Normal Low	5 (7.2) 56 (81.2) 8 (11.6)	6 (9.0) 52 (77.6) 9 (13.4)	0.874
Cd level Normal level < 0.0025 mg/L [24]	High Normal	55 (79.7) 14 (20.3)	63 (92.6) 5 (7.4)	0.028
Co level Normal level < 0.4 mg/L [19]	Normal	69 (100.0)	68 (100.0)	--
Cr level Normal level (0.4–3.5) mg/L [19]	High Normal	0 (0.0) 69 (100.0)	1 (1.5) 67 (98.5)	0.312
Cu level Normal level (0.9–1.5) mg/L [24]	High Normal Low	8 (11.6) 25 (36.2) 36 (52.2)	14 (20.6) 19 (27.9) 35 (51.5)	0.292
Li level Normal level ≤ 13.88 mg/L [25]	Normal	69 (100.0)	68 (100.0)	--
Mg level Normal level (17–24) mg/L [26]	High Normal Low	57 (82.6) 11 (15.9) 1 (1.4)	51 (75.0) 15 (22.1) 2 (2.9)	0.529
Mn level Normal level (0.004–0.015) mg/L [27]	High Normal Low	46 (92.0) 3 (6.0) 1 (2.0)	41(91.1) 4 (8.9) 0 (0.0)	0.557
Ni level Normal level < 0.002 mg/L [28]	High Normal	8 (11.6) 61 (88.4)	4 (5.9) 64 (94.1)	0.237
Pb level Normal level < 0.2 mg/L [24]	High Normal	21 (30.4) 48 (69.6)	15 (22.1) 53 (77.9)	0.265
Sr level Normal level < 0.034 mg/L [29]	High Normal	32 (46.4) 37 (53.6)	31 (45.6) 37 (54.4)	0.926
Zn level Normal level (4.4–8.6) mg/L [24]	High Normal	8 (11.6) 61 (88.4)	10 (14.7) 58 (85.3)	0.590
As level Normal level < 0.012 mg/L [30]	High Normal	20 (29.0) 49 (71.0)	18 (26.5) 50 (73.5)	0.742
Bi level Normal level < 0.05 mg/L [31]	High Normal	42 (61.8) 26 (38.2)	33 (49.3) 34 (50.7)	0.144

* Chi-square test

Al, Aluminum; Se, selenium; V, vanadium; Hg, mercury; Ag, silver; B, boron; Ba, barium; Cd, cadmium; Co, cobalt; Cr, chromium; Cu, copper; Ga, gallium; In, indium; Li lithium; Mg magnesium; Mn, manganese; Ni, nickle; Pb, lead; Sr, strontium; Zn, zinc; As, arsenic; and Bi, bismuth

**Fig. 1:**
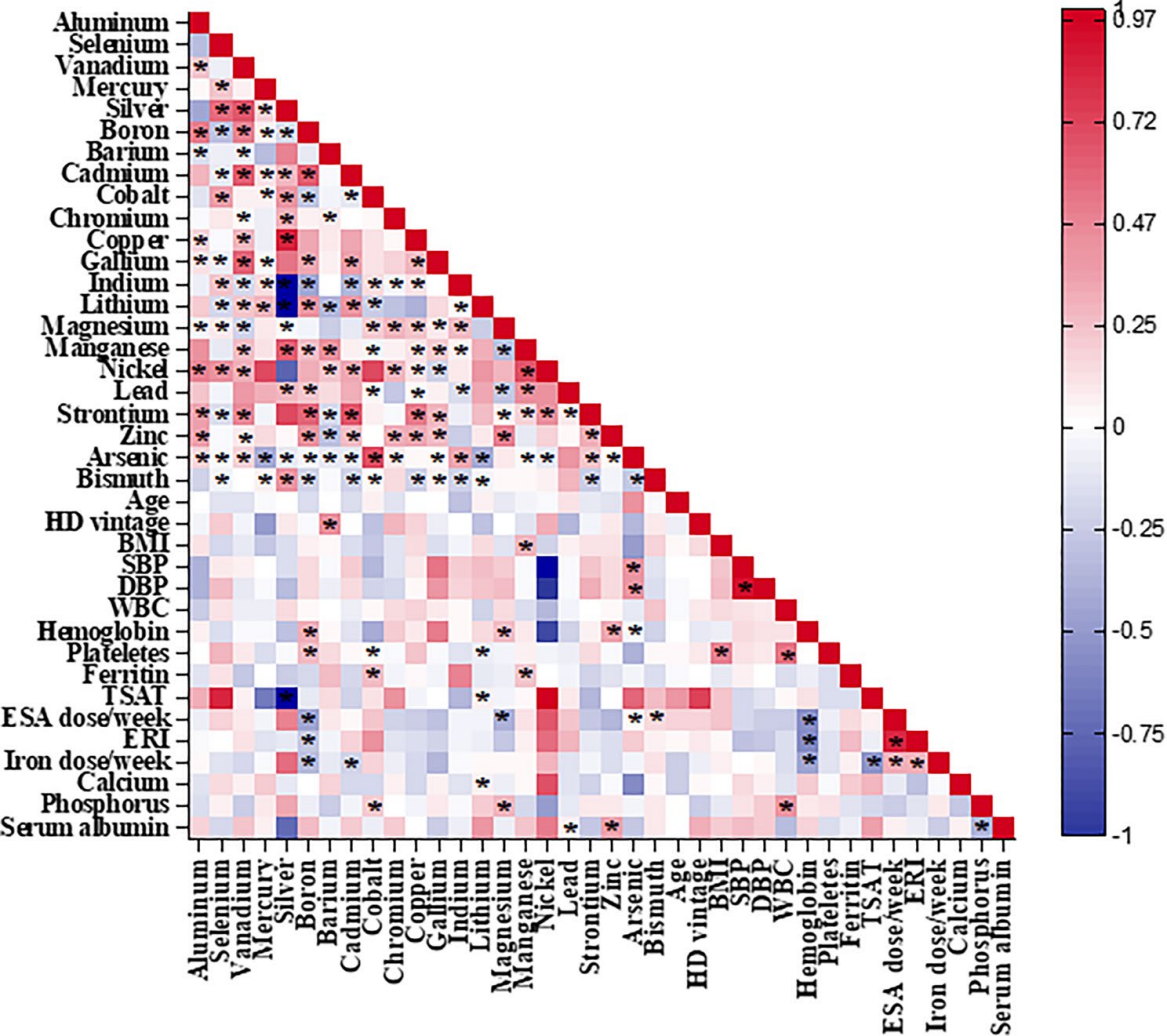
Heatmap represents the correlation matrix among various hematological, biochemical, and trace element parameters in the study cohort. The color gradient indicates the strength and direction of the correlations: red shades denote positive correlations, while blue shades indicate negative correlations. The intensity of the color reflects the magnitude of the correlation coefficient, with darker shades representing stronger associations. Statistically significant correlations (*p* < 0.05) are marked with asterisks (*). BMI, body mass index; DBP, diastolic blood pressure; ERI, erythropoietin resistive index; ESA, erythropoietin stimulating agent; SBP, systolic blood pressure; TSAT, transferrin saturation ratio

A correlation matrix of the study cohort′s hematological, biochemical, and trace element characteristics is shown in the heatmap (Fig. 1). Significant positive correlations were observed between age and Bi (*r* = 0.275, *p* = 0.020); BMI and Mn (*r* = 0.312, *p* = 0.047); HD vintage and Ba (*r* = 0.432, *p* = 0.005); Hb level and B (*r* = 0.321, *p* = 0.012), Mg (*r* = 0.261, *p* = 0.042), Zn (*r* = 0.355, *p* = 0.005), and As (*r* = 0.258, *p* = 0.046); ferritin and Co (*r* = 0.366, *p* = 0.007), and Mn (*r* = 0.273, *p* = 0.044); systolic blood pressure (SBP) and As (*r* = 0.419, *p* = 0.015); diastolic blood pressure (DBP) and As (*r* = 0.391, *p* = 0.024); weekly ESA dose and Bi (*r* = 0.286, *p* = 0.028).

Significant negative correlations were observed between platelets count and Co (*r*=-0.276, *p* = 0.038), and Li (*r*=-0.349, *p* = 0.006); Hb level and ERI (*r*=-0.561, *p* ≤ 0.001); TSAT and Ag (*r*=-0.517, *p* = 0.028); weekly ESA dose and B (*r*=-0.415, *p* = 0.001), Mg (-0.382, *p* = 0.002), and As (*r*=-0.277, *p* = 0.032); I.V. iron dose and Cd (*r*=-0.336, *p* = 0.008), and B (*r*=-0.321, *p* = 0.012); ERI and B (*r*=-0.267, *p* = 0.049).

Multiple linear regression analysis was used to define independent determinants of blood Hb level in HD patients. Zn, Mg, B, and As were incorporated into the model as independent variables. The R2 of the model was 0.070 with *P* = 0.023. The linear regression model revealed that Zn level was an independent predictor of blood Hb level. Furthermore, B, Mg, and As were incorporated into a linear regression model to define the independent predictors of weekly ESA dose. The R2 of the model was 0.128 with *P* = 0.005. The linear regression model revealed that the whole blood level of Mg was an independent predictor of ESA dose per week (Table 8).

**Table 8:** Regression analysis for defining the independent determinants of blood hemoglobin level and weekly erythropoietin dose

Determinants	β	t	*p*-value *
**I. Blood Hb level**			
B (mg/L)	0.051	0.379	0.706
Mg (mg/L)	0.129	0.860	0.393
Zn (mg/L)	0.293	2.337	0.023
Arsenic (mg/L)	0.223	1.791	0.079
**II. ESA dose/week**			
B (mg/L)	-0.159	-0.939	0.352
Mg (mg/L)	-0.358	-2.916	0.005
As (mg/L)	-0.230	-1.912	0.061

* Linear regression analysis

ESA, erythropoietin; Hb, hemoglobin

## Discussion

This study examined whole blood concentrations of 22 trace elements in 137 people, including 69 chronic HD patients and 68 comparison relatives from the same geographical area. HD patients and their comparison relatives had excess blood concentrations of Al, Hg, Ag, Cd, Cu, Mg, Mn, Ni, Pb, Sr, Zn, As, and Bi. In addition, there were significant metal differences across urban and rural populations, hypertensive and non-hypertensive subjects, and diabetics and non-diabetics.

Hemodialysis patients are especially prone to trace elements concentration fluctuations due to reduced renal function and dialysis. Studies have found trace elements deficits and excesses in this group that can affect their clinical results. A comprehensive evaluation of 128 studies found that HD patients have higher levels of cd, Cr, Cu, Pb, and V and lower levels of Se, Zn, and Mn than healthy controls. These trace elements deviated more than 0.8 standard deviation units from control values [32]. Further research showed that HD patients had lower blood levels of Cu, Zn, and Se and greater amounts of Ni, As, and Pb than healthy people [33].

Chronic HD patients also have anomalies in trace elements such as Al, zinc, Mn, and Ni [19]. Several studies stressed the importance of monitoring and managing trace element levels in HD patients to reduce harmful effects and enhance therapeutic outcomes. Our investigations found no significant difference in Al levels between HD patients and their relatives. Prior studies showed that HD patients may accumulate Al due to decreased renal excretion and dialysis-related exposure. Osteomalacia, anemia, and dialysis encephalopathy can result from high serum Al levels. Al toxicity in HD patients was previously caused by dialysis water pollution and Al-based phosphate binders. This population’s serum Al levels have decreased due to water purification improvements and a switch to non-Al binders [34]. Recent studies show that Al exposure remains a risk despite these advances. In Iraq, a study found that HD patients had mean serum Al levels of 21.0 ± 16.0 µg/dL, with 81% exceeding the recommended threshold of 2 µg/dL [35]. Conversely, Taiwanese studies showed a lower mean serum Al level (1.0 ± 0.4 µg/dL), with just 2.2% of patients above the 2 µg/dL. This diversity emphasizes regional monitoring and water quality requirements [36]. To reduce toxicity risk, the National Kidney Foundation’s KDOQI recommends keeping serum aluminum levels below 2 µg/dL in HD patients. Patients with a history of Al exposure, unexplained anemia, or bone disease should have their serum Al levels monitored regularly [17].

This research involved the collection of drinking water samples from two representative sources used by the study population. The samples provided an initial assessment of water quality and potential exposure to trace elements; however, the limited sample size may not sufficiently capture temporal or spatial variability. Future research should include more extensive sampling across diverse locations and time periods. Recent studies indicate that advanced analytical methods, including chemometric modeling and source apportionment techniques such as Positive Matrix Factorization and Monte Carlo Simulation, enhance the accuracy of water quality and risk assessment [37].

Hemodialysis affects serum Mg levels [38]. Humans with normal renal function have tightly regulated serum Mg levels between 0.65 and 1.05 mmol/L. Renal excretion impairment disrupts this equilibrium in HD patients. Research indicates that pre-dialysis serum Mg levels are typically above normal, with mean values of 1.11 ± 0.14 mmol/L and hypermagnesemia in 73.65% of patients. Dialysate Mg concentration affects serum Mg levels, which drop by 0.14 mmol/L post-dialysis [38]. Lower dialysate Mg concentrations (e.g., 0.5 mmol/L) reduce serum Mg levels, while greater values may cause moderate hypermagnesemia [39]. Optimizing patient management and reducing complications requires understanding these consequences. Both hypo- and hypermagnesemia are linked to higher death rates in HD patients, emphasizing the need for proper Mg management [40].

Essential trace element chromium is involved in glucose metabolism [41]. Diet, dialysis equipment infection, and renal clearance can affect HD patients’ serum Cr levels. High serum Cr levels in HD patients are linked to malnutrition and inflammation, suggesting a complex relationship between Cr status and patient health [42]. The effect of HD on serum Cr concentrations is unknown, requiring further research.

A trace element with minimal biological activity, barium can be hazardous at high doses. Few data exist on HD and serum Ba levels. Ba can build up in patients with renal impairment. Hence, HD patients’ barium levels should be monitored and researched to minimize hazardous effects.

This study found elevated whole blood chromium and nickel concentrations in particular subgroups of hemodialysis patients, notably among individuals with industrial occupational exposure. Chromium and nickel are essential trace elements required in minimal concentrations; however, excessive exposure can result in oxidative stress, nephrotoxicity, and a range of systemic effects. Previous studies have defined standard reference ranges for blood chromium and nickel, highlighting their potential toxicological impacts when concentrations exceed physiological thresholds [43–45]. The measured concentrations primarily aligned with established ranges for the general population; however, elevated levels observed in specific occupationally exposed individuals underscore the need for ongoing monitoring and preventive strategies. The findings align with existing literature, indicating that even minor elevations in Cr and Ni may pose risks to individuals with compromised immune systems, such as those suffering from kidney issues.

### Influence of geographical and environmental factors

Urban inhabitants showed greater mean blood Ni levels, whereas rural populations had higher As levels. These discrepancies may be due to setting-specific environmental exposures. Urban industrial operations and vehicle pollution can raise Ni exposure, whereas pesticide-treated agriculture and groundwater contamination in rural areas may increase As levels. A study of metal combinations in urban and rural American populations identified different metal exposure patterns, suggesting different natural and human sources [46]. Rural groundwater As contamination is a major issue. A study of rural Limpopo, South Africa, found that As-contaminated water raised blood As levels [47]. These findings emphasize the importance of geographical and environmental contexts in metal exposure risk assessment. These exposures must be mitigated by tailored public health measures that meet urban and rural needs. Participants living near plastic factories had greater blood Al levels than those living farther away. This confirms that industrial pollution affects trace element exposure and that environmental metal contamination sources contribute to trace element body burdens.

The study found hypertensive participants had lower blood Cr and higher blood Ni and As levels. Diabetics had decreased blood Cr and higher blood Pb levels. This study supports previous research that trace metal abnormalities may contribute to hypertension and diabetes. Diabetics often lack Cr, an essential trace element in glucose metabolism and insulin sensitivity, which may worsen insulin resistance and glucose homeostasis [48, 49]. Both hypertensive and diabetic subjects had reduced blood Cr levels, suggesting a metabolic disruption. In hypertensive and diabetic people, PTEs such as Ni, As, and Pb are higher, supporting the idea that heavy metal exposure may cause cardiometabolic illnesses. Navas-Acien et al. 2008 and Hou et al. 2013 linked As to oxidative stress, endothelial dysfunction, and inflammation, which cause hypertension and diabetes [50, 51]. Ni exposure also raises blood pressure and causes vascular dysfunction, suggesting its role in hypertension [52, 53]. These findings stress the need for more research on trace metal dysregulation and metabolic and cardiovascular health, as well as the need for environmental and dietary strategies to reduce heavy metal exposure in sensitive populations.

Our multivariable logistic regression analysis findings are consistent with recent studies in the literature. Ammar et al. 2023 study showed significant migration of aluminum from cooking pots into food, especially under acidic conditions and high temperature [54]. The toxic-metal content of cigarettes has been reviewed recently, confirming high levels of cadmium, lead, nickel, and other heavy metals in tobacco products which contribute substantially to body burden in smokers [55]. Occupational exposures have similarly been implicated; workers in industrial settings often show elevated vanadium levels, consistent with its known bioavailability and mobility in contaminated soils and emissions [56].

Previous studies in the literature showed that anemia can result from Mg shortage, which is needed for many enzymatic activities, including hemoglobin production [57]. Zn is essential for erythropoiesis and hemoglobin levels. As is hazardous, yet some investigations have found trace levels in human tissues; however, its effect on hematological parameters is unclear and needs further study [58]. The current study’s correlation analysis showed substantial relationships between trace element levels and hematological parameters, suggesting that micronutrients may affect erythropoiesis and anemia therapy. For instance, hemoglobin levels correlated positively with B, Mg, Zn, and As. However, weekly erythropoietin dose negatively correlated with B, Mg, and As. Higher endogenous Mg levels may increase erythropoiesis and reduce the requirement for exogenous erythropoietin (EPO) therapy, as the negative correlation between EPO dose per week and magnesium implies. This is important in chronic renal disease, where EPO is used to treat anemia. Understanding these correlations may help optimize anemia treatment by correcting trace element shortages through nutritional interventions. These findings should be interpreted cautiously because correlation does not imply causality, and more research is needed to understand the mechanisms and make treatment recommendations.

Recent studies highlight a persistent deficiency of essential trace elements such as Se in HD patients, contrasted by accumulation of PTEs such as Mn, Pb, and Cd. A meta-analysis of randomized controlled trials (RCTs) confirmed that Se supplementation significantly increases plasma Se levels in this population, although it failed to produce meaningful improvements in lipid profiles, inflammation, or hemoglobin levels [59]. A recent study of erythrocytes from ESRD patients found significantly reduced Fe and Zn, yet increased Mn, Li, Pb, and Cd, compared to healthy controls highlighting impaired excretion and potential intracellular burdens even when serum levels might be unremarkable [60]. In a multicenter study from Jordan, HD patients exhibited markedly higher blood Pb, Cd, and Cu and lower Zn levels than healthy controls, with age-related accumulation patterns suggesting cumulative exposure risks [61]. Meanwhile, Trigueira et al. 2024 systematic review and meta-analysis reaffirmed that Se supplementation effectively restores plasma Se but yields inconsistent effects on antioxidant, inflammatory, and thyroid parameters [62].

Looking forward, the field is poised to transition toward multi-element profiling using advanced techniques such as Inductively Coupled Plasma Mass Spectrometry (ICP-MS) across various biological compartments e.g., (whole blood, and erythrocytes), to enhance detection of both deficiency and toxic accumulation. Such comprehensive monitoring will lay the groundwork for personalized supplementation strategies, targeting individual trace-element imbalances. At the same time, the tightening of international standards for dialysis water quality will reduce inadvertent exposure to harmful elements. Together, these developments indicate a paradigm shift from descriptive characterization toward integrated, outcome-oriented management, with the potential to improve clinical outcomes in dialysis patients.

This study has several limitations that should be acknowledged. First, it was conducted in a single-center setting with a relatively limited sample size, which may restrict the generalizability of the findings. In addition, dietary intake a potential confounding factor, was not comprehensively evaluated, which could have influenced trace-element levels. The cross-sectional design also precludes establishing causality between the observed disturbances and clinical outcomes. Despite these limitations, the study has notable strengths. To our knowledge, this study is among few studies that provide a comprehensive multi-element analysis in HD patients, including both essential and toxic trace elements. Moreover, the use of ICP-OES for whole-blood assessment adds methodological rigor and enhances sensitivity compared to conventional serum-only assessments. This investigation was also limited by the sampling of only two water sources. This complicates the application of water quality data across various sources and may not accurately reflect trace element concentrations over time and across different locations. The role of drinking water in trace element intake warrants careful consideration. Future research should employ broad, temporally stratified samples alongside advanced statistical and chemometric techniques to investigate water quality and health.

## Conclusion

This study demonstrates marked disturbances in trace element balance among HD patients, with evidence of deficiencies in essential elements and accumulation of PTEs. Using ICP-OES, we provide a comprehensive multi-element profile that highlights the dual burden of deficiency and overload. This work offers important new insights into the intricate relationship that exists between the concentrations of trace elements, the environmental exposures, and the health condition of HD patients that underscoring the need for region-specific monitoring and preventive strategies. Clinically, our results reinforce the importance of incorporating trace element assessment into the routine care of HD patients and developing targeted supplementation protocols. Looking forward, future research should focus on multicenter, longitudinal studies that combine sensitive analytical platforms (such as ICP-MS) with clinical outcome measures. In addition, future efforts should aim to evaluate and modify environmental risk factors, alongside optimizing dialysis water quality standards, to reduce inadvertent trace elements exposure. Such strategies are expected to pave the way for evidence-based, personalized interventions that improve long-term outcomes in the HD population.

## Supplementary Information

Below is the link to the electronic supplementary material.

Supplementary Material 1

## Data Availability

This article and its supplementary material files include all data generated or analyzed during this study. Further enquiries can be directed to the corresponding author.

## References

[1] Bello AK, Okpechi IG, Levin A, Ye F, Damster S, Arruebo S, et al. An update on the global disparities in kidney disease burden and care across world countries and regions. Lancet Global Health. 2024;12(3):e382–95. 38365413 10.1016/S2214-109X(23)00570-3

[2] Bello AK, Levin A, Lunney M, Osman MA, Ye F, Ashuntantang GE, et al. Status of care for end stage kidney disease in countries and regions worldwide: international cross sectional survey. BMJ. 2019;367. 10.1136/bmj.l5873 31672760

[3] Tonelli M, Wiebe N, Bello A, Field CJ, Gill JS, Hemmelgarn BR, et al. Concentrations of trace elements and clinical outcomes in Hemodialysis patients: a prospective cohort study. Clin J Am Soc Nephrol. 2018;13(6):907–15. 29599300 10.2215/CJN.11451017 PMC5989679

[4] López-Alonso M, Rivas I, Miranda M. Trace mineral imbalances in global health: Challenges, Biomarkers, and the role of serum analysis. Nutrients. 2025;17(13):2241. 40647345 10.3390/nu17132241 PMC12251835

[5] Abd Alati AE, Abd A-HAE-M, El-Hamid SAE-H, Yousef DM, Abd El-Gawad ER, Zakaria RM. Serum level of trace elements in pediatric patients with end stage renal disease on Hemodialysis. Egypt J Hosp Med (April. 2024;95:2134–41.

[6] El-Shazly AN, Ibrahim SAE-H, El-Mashad GM, Sabry JH, Sherbini NS. Effect of zinc supplementation on body mass index and serum levels of zinc and leptin in pediatric Hemodialysis patients. Int J Nephrol Renovascular Disease. 2015:159–63. 10.2147/IJNRD.S94923 PMC4677656 26677341

[7] Wang L-J, Wang M-Q, Hu R, Yang Y, Huang Y-S, Xian S-X, et al. Effect of zinc supplementation on maintenance Hemodialysis patients: A systematic review and meta-analysis of 15 randomized controlled trials. Biomed Res Int. 2017;2017(1):1024769. 29457023 10.1155/2017/1024769 PMC5804106

[8] Kobayashi H, Abe M, Okada K, Tei R, Maruyama N, Kikuchi F, et al. Oral zinc supplementation reduces the erythropoietin responsiveness index in patients on Hemodialysis. Nutrients. 2015;7(5):3783–95. 25988769 10.3390/nu7053783 PMC4446779

[9] King KE, Darrah TH, Money E, Meentemeyer R, Maguire RL, Nye MD, et al. Geographic clustering of elevated blood heavy metal levels in pregnant women. BMC Public Health. 2015;15:1–12. 26449855 10.1186/s12889-015-2379-9 PMC4599656

[10] Zaynab M, Al-Yahyai R, Ameen A, Sharif Y, Ali L, Fatima M, et al. Health and environmental effects of heavy metals. J King Saud University-Science. 2022;34(1):101653.

[11] Huang W-H, Hu C-C, Yen T-H, Hsu C-W, Weng C-H. Blood lead level: an overlooked risk of carpal tunnel syndrome in Hemodialysis patients. Ren Fail. 2019;41(1):786–93. 31498017 10.1080/0886022X.2019.1657894 PMC6746292

[12] Humudat YR, Al-Naseri SK. Heavy metals in Dialysis fluid and blood samples from Hemodialysis patients in Dialysis centers in baghdad, Iraq. J Health Pollution. 2020;10(27):200901. 10.5696/2156-9614-10.27.200901 PMC7453809 32874757

[13] Anyanwu BO, Ezejiofor AN, Igweze ZN, Orisakwe OE. Heavy metal mixture exposure and effects in developing nations: an update. Toxics. 2018;6(4):65. 30400192 10.3390/toxics6040065 PMC6316100

[14] Mortada WI, Awadalla A, Khater S, Ahmed A, Hamam ET, El-Zayat M, et al. Copper and zinc levels in plasma and cancerous tissues and their relation with expression of VEGF and HIF-1 in the pathogenesis of muscle invasive urothelial bladder cancer: a case-controlled clinical study. Environ Sci Pollut Res. 2020;27:15835–41. 10.1007/s11356-020-08113-8 32095963

[15] Organization WH. Guidelines for drinking-water quality: incorporating the first and second addenda. World Health Organization; 2022. 35417116

[16] Instrumentation AftAoM. ANSI/AAMI/ISO 23500: 2011 guidance for the Preparation and quality management of fluids for Hemodialysis and related therapies. Arlingt VA. 2011:9–30.

[17] Eknoyan G, Levin A, Levin NW. Bone metabolism and disease in chronic kidney disease. Am J Kidney Dis. 2003;42:1–201.

[18] Stockler-Pinto MB, Malm O, Azevedo SRG, Farage NE, Dorneles PR, Cozzolino SMF, et al. Selenium plasma levels in Hemodialysis patients: comparison between North and Southeast of Brazil. Jornal Brasileiro De Nefrologia. 2014;36(4):490–5. 25517278 10.5935/0101-2800.20140070

[19] Wakino S. Trace elements and their management in Dialysis Patients—Pathophysiology and clinical manifestations. Kidney Dialysis. 2023;3(3):274–96.

[20] Ye B-J, Kim B-G, Jeon M-J, Kim S-Y, Kim H-C, Jang T-W, et al. Evaluation of mercury exposure level, clinical diagnosis and treatment for mercury intoxication. Annals Occup Environ Med. 2016;28:1–8. 10.1186/s40557-015-0086-8 PMC4724159 26807265

[21] Steck MB, Murray BP. Silver toxicity. StatPearls [Internet]: StatPearls Publishing; 2024. 38861631

[22] Moseman RF. Chemical disposition of Boron in animals and humans. Environ Health Perspect. 1994;102(suppl 7):113–7. 7889870 10.1289/ehp.94102s7113 PMC1566637

[23] Moffett D, Smith-Simon C, Stevens Y-W. Toxicological profile for barium and barium compounds. 2007. 38147518

[24] Lee SH, Huang JW, Hung KY, Leu LJ, Kan YT, Yang CS, et al. Trace metals’ abnormalities in Hemodialysis patients: relationship with medications. Artif Organs. 2000;24(11):841–4. 11119069 10.1046/j.1525-1594.2000.06352.x

[25] Hedya SA, Avula A, Swoboda HD. Lithium toxicity. StatPearls [Internet]: StatPearls Publishing; 2023. 29763168

[26] Alhosaini M, Leehey DJ. Magnesium and dialysis: the neglected cation. Am J Kidney Dis. 2015;66(3):523–31. 25864370 10.1053/j.ajkd.2015.01.029

[27] Khan M, Orakzai SA, Sarwar A, Shah W, Ali A. Estimation of blood manganese (trace element) levels in patients of hepatitis C, cirrhosis and hepatocellular carcinoma. Adv Basic Med Sci. 2022;6(1):3–7.

[28] Alimonti A, Bocca B, Mattei D, Pino A. Programme for biomonitoring the Italian population exposure (PROBE): internal dose of metals. 2011.

[29] Amata R, Diamond GL, Dorsey A, Fransen ME. Toxicological profile for strontium. 2004.

[30] Hall M, Chen Y, Ahsan H, Slavkovich V, Van Geen A, Parvez F, et al. Blood arsenic as a biomarker of arsenic exposure: results from a prospective study. Toxicology. 2006;225(2–3):225–33. 16860454 10.1016/j.tox.2006.06.010

[31] Çelebi-Tayfur A, Yaradılmış RM, Ulus F, Çaltık-Yılmaz A, Özayar E, Koşar B, et al. Bismuth intoxication resulting in acute kidney injury in a pregnant adolescent Girl. Turk J Pediatr. 2019;61(2):292–6. 31951346 10.24953/turkjped.2019.02.024

[32] Tonelli M, Wiebe N, Hemmelgarn B, Klarenbach S, Field C, Manns B, et al. Trace elements in Hemodialysis patients: a systematic review and meta-analysis. BMC Med. 2009;7:1–12. 19454005 10.1186/1741-7015-7-25 PMC2698829

[33] Azevedo R, Gennaro D, Duro M, Pinto E, Almeida A. Further evidence on trace element imbalances in haemodialysis Patients—Paired analysis of blood and serum samples. Nutrients. 2023;15(8):1912. 37111132 10.3390/nu15081912 PMC10145991

[34] Ryan T, McElwain L, Murphy T, Arduino M, Anderson S. Elevated serum aluminum levels in hemodialysis patients associated with use of electric pumps–wyoming, 2007. MMWR: Mortality Weekly Report. 2008;57(25). 18583956

[35] Humudat YR, Al-Naseri SK. Heavy metals in dialysis fluid and blood samples from hemodialysis patients in dialysis centers in baghdad, Iraq. J Health Pollution. 2020;10(27). 10.5696/2156-9614-10.27.200901 PMC7453809 32874757

[36] Chuang P-H, Tsai K-F, Wang I-K, Huang Y-C, Huang L-M, Liu S-H, et al. Blood aluminum levels in patients with Hemodialysis and peritoneal Dialysis. Int J Environ Res Public Health. 2022;19(7):3885. 35409569 10.3390/ijerph19073885 PMC8997989

[37] Ustaoğlu F, Yüksel B, Yazman MM, Jaskuła J, Tokatlı C. Chemometric investigation of river system contamination: source identification and risk assessment using positive matrix factorization and Monte Carlo simulation. J Contam Hydrol. 2025:104627. 10.1016/j.jconhyd.2025.104627 40440967

[38] Han Z, Zhou L, Liu R, Feng L. The effect of Hemodialysis on serum magnesium concentration in Hemodialysis patients. Annals Palliat Med. 2020;9(3):1134143–1143. 10.21037/apm-20-992 32498528

[39] Cunningham J, Rodríguez M, Messa P. Magnesium in chronic kidney disease stages 3 and 4 and in Dialysis patients. Clin Kidney J. 2012;5(Suppl1):i39–51. 26069820 10.1093/ndtplus/sfr166 PMC4455820

[40] Pérez-García R, Jaldo MT, Puerta M, Ortega M, Corchete E, de Sequera P, et al. Hypomagnesemia in Hemodialysis is associated with increased mortality risk: its relationship with Dialysis fluid. Nefrología (English Edition). 2020;40(5):552–62. 10.1016/j.nefro.2020.04.013 32651086

[41] Zhao F, Pan D, Wang N, Xia H, Zhang H, Wang S, et al. Effect of chromium supplementation on blood glucose and lipid levels in patients with type 2 diabetes mellitus: a systematic review and meta-analysis. Biol Trace Elem Res. 2022:1–10. 10.1007/s12011-021-02693-3 33783683

[42] Hsu C-W, Weng C-H, Lee C-C, Yen T-H, Huang W-H. Association of serum chromium levels with malnutrition in Hemodialysis patients. BMC Nephrol. 2019;20:1–9. 31382911 10.1186/s12882-019-1476-x PMC6683568

[43] Yüksel B, Arıca E, Söylemezoğlu T. Assessing reference levels of nickel and chromium in cord blood, maternal blood and placenta specimens from Ankara, Turkey. J Turkish German Gynecol Association. 2021;22(3):187. 10.4274/jtgga.galenos.2021.2020.0202 PMC8420753 33631873

[44] Yüksel B, Kayaalti Z, Söylemezoglu T, Türksoy VA, Tutkun E. GFAAS determination of arsenic levels in biological samples of workers occupationally exposed to metals: an application in analytical toxicology. At Spectrosc. 2015;36(4):171–6.

[45] Yüksel B, Kaya S, Kaya-Akyüzlü D, Kayaalti Z, Soylemezoglu T. Validation and optimization of an analytical method based on cold vapor atomic absorption spectrometry for the determination of mercury in maternal blood, cord blood, and placenta samples. Spectrosc. 2017;38(4):112–6.

[46] Pang Y, Peng RD, Jones MR, Francesconi KA, Goessler W, Howard BV, et al. Metal mixtures in urban and rural populations in the US: the Multi-Ethnic study of atherosclerosis and the strong heart study. Environ Res. 2016;147:356–64. 26945432 10.1016/j.envres.2016.02.032 PMC4827253

[47] Kapwata T, Wright CY, Reddy T, Street R, Kunene Z, Mathee A. Relations between personal exposure to elevated concentrations of arsenic in water and soil and blood arsenic levels amongst people living in rural areas in Limpopo, South Africa. Environ Sci Pollut Res. 2023;30(24):65204–16. 10.1007/s11356-023-26813-9 PMC10116462 37079235

[48] Vincent J. The nutritional biochemistry of chromium (III). Elsevier; 2018.

[49] Cefalu W, Hu F. Role of chromium in human health and in diabetes. Diabetes Care. 2004; 27: 2741–2751. Diabetes Care. 2013;36(9):2872. 10.2337/diacare.27.11.2741 15505017

[50] Navas-Acien A, Silbergeld EK, Pastor-Barriuso R, Guallar E. Arsenic exposure and prevalence of type 2 diabetes in US adults. JAMA. 2008;300(7):814–22. 18714061 10.1001/jama.300.7.814

[51] Balakumar P, Kaur T, Singh M. Potential target sites to modulate vascular endothelial dysfunction: current perspectives and future directions. Toxicology. 2008;245(1–2):49–64. 18242815 10.1016/j.tox.2007.12.011

[52] Liu Y, Wu M, Xu B, Kang L. Association between the urinary nickel and the diastolic blood pressure in general population. Chemosphere. 2022;286:131900. 34411926 10.1016/j.chemosphere.2021.131900

[53] Cheek J, Fox SS, Lehmler H-J, Titcomb TJ. Environmental nickel exposure and cardiovascular disease in a nationally representative sample of US adults. Exposure Health. 2024;16(2):607–15. 10.1007/s12403-023-00579-4 PMC10249564 37360515

[54] Ammar HR, Saleh SM, Sivasankaran S, Albadri AE, Al-Mufadi FA. Investigation of element migration from aluminum cooking pots using ICP-MS. Appl Sci. 2023;13(24):13119.

[55] Felix AT, Ntarisa AV. Review of toxic metals in tobacco cigarette brands and risk assessment. J King Saud University-Science. 2024;36(10):103484.

[56] Wnuk E. Mobility, bioavailability, and toxicity of vanadium regulated by physicochemical and biological properties of the soil. J Soil Sci Plant Nutr. 2023;23(1):1386–96.

[57] Huang J, Xu J, Ye P, Xin X. Association between magnesium intake and the risk of anemia among adults in the united States. Front Nutr. 2023;10:1046749. 36908911 10.3389/fnut.2023.1046749 PMC9996106

[58] Abernathy CO, Liu Y-P, Longfellow D, Aposhian HV, Beck B, Fowler B, et al. Arsenic: health effects, mechanisms of actions, and research issues. Environ Health Perspect. 1999;107(7):593–7. 10379007 10.1289/ehp.99107593 PMC1566656

[59] Cheng Q, Fan D, Chen P, Yuan H. Effect of selenium supplementation on hemodialysis patients: a meta-analysis. Int Urol Nephrol. 2025:1–9. 10.1007/s11255-025-04400-w 39900786

[60] Rajkowska-Myśliwiec M, Szczuko M, Witczak A, Kaczkan M, Małgorzewicz S. Assessment of essential and toxic trace element levels in erythrocytes of Hemodialysis patients with end-stage renal disease. J Trace Elem Med Biol. 2024;85:127491. 38943837 10.1016/j.jtemb.2024.127491

[61] Orabi FMA, Fawzi M, Humaidi J, Shammout M-J, Saleh R, Hakawati H, et al. Heavy metal blood levels and their age-dependent changes in patients undergoing Hemodialysis at Jordan Islamic hospital. Ukrainian J Nephrol Dialysis. 2024;3(83):33–40.

[62] Trigueira PC, Cardoso LVO, Mafra BR, Araujo D, Stockler-Pinto MC. MB. Selenium supplementation in chronic kidney disease patients undergoing haemodialysis: a systematic review of the effects on plasma selenium, antioxidant and inflammatory markers, immunological parameters and thyroid hormones. Nutr Res Rev. 2024:1–12. 10.1017/S0954422424000222 39320843

