# Supplementary material for: Comparative analysis of blood trace elements in Egyptian hemodialysis patients and their relatives in the same geographical area, is dialysis still guilty?
Source: BMC Nephrol. 2025 Dec 12;27:47. doi: 10.1186/s12882-025-04636-9 (PMC12817547; doi:10.1186/s12882-025-04636-9)
Supplement: Supplementary file 1 — Supplementary Material 1 [file 12882_2025_4636_MOESM1_ESM.docx]

**Table S1:** Approximate Median and IQR of Whole Blood Trace Elements (mg/L).

| **Trace element** | **HD** | | **Control** | |
| --- | --- | --- | --- | --- |
|  | **Median** | **IQR (Q1–Q3)** | **Median** | **IQR (Q1–Q3)** |
| **Al**  **Se**  **V**  **Hg**  **Ag**  **B**  **Ba**  **Cd**  **Co**  **Cr**  **Cu**  **Ga**  **In**  **Li**  **Mg**  **Mn**  **Ni**  **Pb**  **Sr**  **Zn**  **As**  **Bi** | 0.08  0.12  0.08  0.02  0.03  1.50  0.18  0.03  0.02  2.05  0.70  0.04  0.80  0.06  34.00  0.04  0.01  0.10  0.02  6.50  0.00  0.15 | 0.02 – 0.18  0.04 – 0.25  0.02 – 0.17  0.00 – 0.05  0.00 – 0.08  0.60 – 5.20  0.10 – 0.30  0.02 – 0.06  0.01 – 0.05  1.70 – 2.55  0.35 – 1.10  0.01 – 0.15  0.30 – 2.00  0.02 – 0.18  27.00 –44.50  0.03 – 0.05  0.00 – 0.03  0.05 – 0.20  0.01 – 0.05  4.50 – 9.50  0.00 – 0.01  0.05 – 0.40 | 0.05  0.13  0.09  0.01  0.02  1.80  0.14  0.03  0.02  2.10  0.75  0.03  0.75  0.04  30.50  0.04  0.03  0.08  0.03  5.90  0.00  0.12 | 0.01 – 0.12  0.05 – 0.28  0.02 – 0.19  0.00 – 0.03  0.00 – 0.06  0.70 – 6.50  0.08 – 0.25  0.02 – 0.06  0.01 – 0.06  1.80 – 2.80  0.40 – 1.20  0.00 – 0.20  0.25 – 1.90  0.01 – 0.12  25.00 – 38.00  0.03 – 0.05  0.01 – 0.10  0.04 – 0.15  0.01 – 0.05  4.20 – 8.00  0.00 – 0.01  0.04 – 0.30 |

**Table S2:** Metals and trace elements concentrations in drinking tap water in two locations of participants' residency in comparison with WHO standards (1).

| **Trace element** | **Location 1**  **(mg/L)** | **Location 2**  **(mg/L)** | **WHO regulatory standard**  **(mg/L)** |
| --- | --- | --- | --- |
| **Al**  **Se**  **V**  **Hg**  **Ag**  **B**  **Ba**  **Cd**  **Co**  **Cr**  **Cu**  **Ga**  **In**  **Li**  **Mg**  **Mn**  **Ni**  **Pb**  **Sr**  **Zn**  **As**  **Bi** | ND  ND  ND  ND  ND  2.14  ND  0.002  ND  ND  0.030  ND  ND  ND  1.12  0.006  0.02  0.01  0.026  0.02  ND  ND | ND  0.024  ND  ND  ND  2.01  ND  0.001  0.001  ND  0.035  ND  ND  ND  1.16  0.007  0.03  ND  0.026  0.02  ND  ND | 0.2  0.05  --  0.001  0.001  --  --  0.003  --  0.05  2  --  --  --  --  0.4  0.07  0.05  --  3  0.01  -- |

**Table S3:** Metals and trace elements concentrations in dialysate water in comparison with Association for the Advancement of Medical Instrumentation (AAMI) maximum allowable concentrations (2).

| **Trace element** | **Before reverse osmosis sample (mg/L)** | **Final dialysis water sample (mg/L)** | **AAMI* Maximum allowable Concentration (mg/L)** |
| --- | --- | --- | --- |
| **Al**  **Se**  **V**  **Hg**  **Ag**  **B**  **Ba**  **Cd**  **Co**  **Cr**  **Cu**  **Ga**  **In**  **Li**  **Mg**  **Mn**  **Ni**  **Pb**  **Sr**  **Zn**  **As**  **Bi** | ND  ND  0.004  ND  ND  2.19  ND  ND  0.014  ND  0.038  ND  ND  ND  0.06  0.008  0.03  0.014  0.001  0.008  ND  ND | ND  ND  ND  ND  ND  2.17  ND  ND  0.005  ND  0.028  ND  ND  ND  0.04  0.005  0.031  0.019  0.002  0.051  ND  ND | 0.01  0.09  --  0.0002  0.005  --  0.1  0.001  --  0.014  0.1  --  --  --  4  --  --  0.005  --  0.1  0.005  -- |

AAMI=Association for the Advancement of Medical Instrumentation.

**References:**

1. Organization WH. Guidelines for drinking-water quality: incorporating the first and second addenda: World Health Organization; 2022.

2. Instrumentation AftAoM. ANSI/AAMI/ISO 23500: 2011 Guidance for the preparation and quality management of fluids for hemodialysis and related therapies. Arlington VA. 2011:9-30.
